# Cooperative foraging expands dietary niche but does not offset intra-group competition for resources in social spiders

**DOI:** 10.1038/s41598-018-30199-x

**Published:** 2018-08-07

**Authors:** Marija Majer, Christina Holm, Yael Lubin, Trine Bilde

**Affiliations:** 1 Blaustein Institutes for Desert Research, Mitrani Department of Desert Ecology, Ben-Gurion University of the Negev, Midreshet Ben-Gurion, 8499000 Israel; 2 Institute of Bioscience, Aarhus University, Ny Munkegade 114, 8000 Aarhus, Denmark

## Abstract

Group living animals invariably risk resource competition. Cooperation in foraging, however, may benefit individuals in groups by facilitating an increase in dietary niche. To test this, we performed a comparative study of social and solitary spider species. Three independently derived social species of *Stegodyphus* (Eresidae) occupy semi-arid savannas and overlap with three solitary congeners. We estimated potential prey availability in the environment and prey acquisition by spiders in their capture webs. We calculated dietary niche width (prey size) and breadth (taxonomic range) to compare resource use for these six species, and investigated the relationships between group size and average individual capture web production, prey biomass intake rate and variance in biomass intake. Cooperative foraging increased dietary niche width and breadth by foraging opportunistically, including both larger prey and a wider taxonomic range of prey in the diet. Individual capture web production decreased with increasing group size, indicating energetic benefits of cooperation, and variance in individual intake rate was reduced. However, individual biomass intake also decreased with increasing group size. While cooperative foraging did not completely offset resource competition among group members, it may contribute to sustaining larger groups by reducing costs of web production, increasing the dietary niche and reducing the variance in prey capture.

## Introduction

Inter-specific competition for resources plays an important role in the evolution of foraging adaptations and the ecological niche of individuals and species, and may ultimately drive adaptive speciation^1–4^. Interspecific competition exists at all trophic levels, but where herbivores are likely to be limited by their predators rather than resources, predator populations are more likely to be limited by their prey^5^. Resource competition can be mitigated by the evolution of foraging strategies that modify the way resources are obtained^6^. Cooperative foraging may function to change a species niche to exploit resources unavailable to individual foragers^7^, or facilitate more efficient exploitation of resources^8,9^. The economics of social foraging depend on the dynamics of food discovery^8^, and the means by which food is acquired, for example by the use of coordinated hunting strategies that enhance prey capture (e.g. in lions)^10^, or the evolution of specialized food gathering castes as in the eusocial insects^11^. In sedentary group foragers, such as social spiders, that are unable to forage outside the nest and associated capture web, cooperative strategies that involve building larger communal webs and collective hunting of insect prey may be particularly important for sustaining the group^12–15^.

There are several ways by which cooperative foraging may afford foraging benefits. Group hunting can result in increased prey capture rate^12,16^, or enable an increase of dietary niche^17–20^. The latter may arise from inclusion of larger prey to widen the dietary niche^16,17,21^, or through an increase in diet breadth by the inclusion of a broader taxonomic range of food or prey types in the diet^22–24^. This could lead to an expansion in resource use or a shift in diet to exploit a different dietary niche^7,25–27^. Group foraging can also provide benefits by reducing variance in prey capture rate and individual consumption rate, which buffers the group against starvation^28–30^. Finally, cooperative foraging can provide energetic benefits by reducing the individual investment in resource acquisition^31^. These potential benefits are not mutually exclusive.

Cooperation in spiders has evolved independently multiple times, with approximately 20 origins in seven families out of the more than 46,000 known extant species^32–36^. Social spiders share a communal nest, where they cooperate in web building and prey capture, and breed cooperatively^34^. The permanently social species occur in tropical and subtropical areas that are also characterized by higher productivity and prey biomass^15,20,37^, suggesting that food availability or benefits of cooperative foraging are involved in the evolution or maintenance of permanent sociality. Prey size was shown to play an important role in predicting the elevational distribution of social spiders of the new world spider genus *Anelosimus*^37–39^. Social *Anelosimus* species were found to catch larger prey than solitary congeners^40,41^, and prey size distributions differed between the lowland tropical habitat of social *A*. *eximius* and higher elevation solitary species. Larger insects were found in the lowland habitat of social *Anelosimus*^20–22^, supporting the hypothesis that elevational differences in the size of insect prey may explain the geographical distribution of these *Anelosimus* species. In addition, a study of the social *A*. *eximius* and subsocial (solitary) *A*. *baeza* suggested that the social species exploit a wider range of insect types from their environment^22^. Collectively there is good support for exploitation of larger prey, i.e. a wider dietary niche, in cooperatively foraging *Anelosimus*, which is associated with their occurrence in lowland tropical areas with higher frequency of large prey (18, 29, and references above). Whether this is a common pattern in other social spider species, however, remains unknown.

Group living invariably comes with competition for resources among group members that limits breeding opportunities^42,43^, and competition for resources is expected to increase with group size^9^. Both the costs and the benefits of group living are often positively correlated with group size, imposing opposing selective forces on group size^44^. Social spiders build large capture webs, and individuals may acquire energetic benefits from reduced investment in silk production and web construction with increasing group size^31,45^. A study of the South American *A*. *eximius* showed that the surface/volume ratio of the three-dimensional capture web decreased with increasing group size, supporting this hypothesis^13^. However, prey capture per individual declined with increasing group size, and although larger groups succeeded in capturing larger prey^46^, individual biomass intake was maximized at an intermediate group size^13^. In *A*. *eximius* and the African social spider *Stegodyphus dumicola*, colony survival increased with increasing group size^47,48^, yet for both species, individual lifetime reproductive success was highest at intermediate group sizes, suggesting that competition for resources within the colony limits group size^47,48^. These studies, as well as theoretical considerations^49,50^, suggest that the interaction between group size and individual resource acquisition plays an important role in the maintenance of sociality.

Here we investigated the hypothesis that cooperative foraging enables groups to increase the range of resources they can obtain from the environment, by comparing the foraging strategies of congeneric social and solitary spiders in relation to resource availability in the habitat. We present results from a comprehensive, comparative study of six species of the Old-World genus *Stegodyphus* (Eresidae), in which we investigated effects of cooperative foraging on resource acquisition, as measured by the dietary niche width and breadth, and potential energetic benefits of silk saving in producing a group capture web. The genus *Stegodyphus* contains three independent origins of cooperative social species^35,51^. These social species occur in areas characterized by higher productivity and insect abundance compared with solitary congeners^52^, but unlike the New-World genus *Anelosimus*, there is no pronounced elevational gradient, as both social and solitary species typically occur in low- to mid-elevation sub-tropical and semi-arid regions. To test the hypothesis that social *Stegodyphus* avail themselves of a broader foraging niche, we compared resource use in three social and three solitary *Stegodyphus* species. Using traps to estimate prey availability in the habitat, and determining the prey captured and exploited by spiders, we obtained quantitative estimates of dietary niche width (prey size) and breadth (taxonomic range) in relation to potential prey in the nearby habitat^1,19^.

To assess the potential collective benefits arising from prey biomass acquisition in relation to web size at the individual level, we determined capture web sizes in relation to group size and foraging type (social versus solitary), and estimated per capita biomass consumption. We compared these data *between* social and solitary species to assess benefits of cooperative foraging, and *within* the three social *Stegodyphus* species to examine how individual intake rate interacts with group size to offset intraspecific resource competition.

We addressed the following questions:

i. Does cooperative foraging facilitate an expansion in dietary niche width by opportunistic feeding on a wider range of prey sizes, or by a shift in resource use through specialization on larger prey? Dietary niche width was quantified based on the variation in resource use (prey size) within individuals or social groups (WIC) and in the total population (TNW), corresponding to Roughgarden’s within-individual and total population components of niche width, and compared between social and solitary foragers.
ii. Does cooperative foraging enable an expansion (or shift) in dietary niche breadth, i.e., do social species forage on a wider taxonomic range of prey types than solitary foragers? Dietary niche breadth was determined as the taxonomic spectrum of insect prey included in the diet relative to potential prey in the local environment and compared between social and solitary foragers.
iii. How do group living and group size influence individual production of capture web? Capture web used for interception of prey is costly to produce^53^; therefore, cooperative foraging may provide energetic benefits by reduced individual production of silk. We compared individual capture web size in social and solitary foragers, and in relation to group size in social species.
iv. How do cooperative foraging and group size influence individual consumption rate? We determined prey capture in relation to group size, and estimated individual intake rate (mg biomass prey/hour/web cm^2^/ individual) using taxon-specific relationships between insect prey size and mass, comparing social and solitary foraging mode.
v. How do potential benefits of cooperative foraging interact with group size to offset intra-specific competition? We examined the relationship between group size, individual prey capture rate and web size within the three social *Stegodyphus* species. Finally, we assessed the effect of group size on variance in individual intake rate.

## Materials and Methods

### Study species

**Figure 1:**
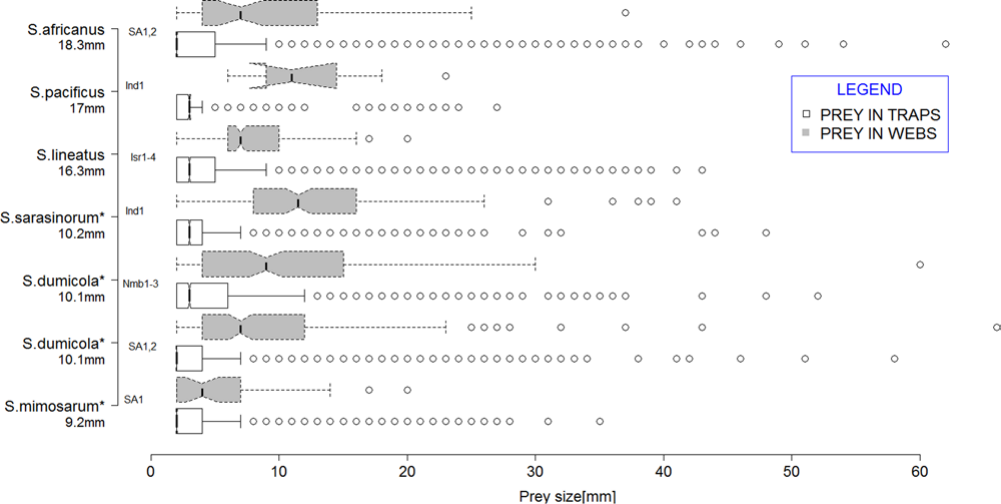
Boxplots of available (*white*) and consumed prey size (*grey*) in the diets of three solitary and three social *Stegodyphus* species. Data are plotted for solitary *S*. *africanus S*. *lineatus* and *S*. *pacificus*, followed by social *S*. *dumicola*, *S*. *mimosarum* and *S*. *sarasinorum* (social species marked with*). Numbers on the y-axis below the species names show the average body size of adult female spiders. Abbreviations next to the boxplots represent sites (see Table 2), where species data collected at different sites are combined. Each boxplot shows the extremes, the inter-quartile range, and the median.

We compared three solitary species and three social species in the genus *Stegodyphus* (Eresidae) (Table 1). *Stegodyphus* species construct nests that consist of silk and plant material on shrubs or trees, either solitarily or in social groups^54^. All species construct aerial sheet-webs consisting of non-sticky threads radiating from the nest and inter-connected with sticky, cribellate silk threads that serve to trap flying and jumping insects^51,55^. Capture webs of both solitary and social species consist of one or more such planar webs. Females produce a single egg-sac; the young are protected and fed by their mother by regurgitation feeding, and females are eventually consumed by the offspring^54,56^. In the solitary species, the young disperse out of the maternal nest, while in the social species, offspring remain in the maternal nest where they capture prey cooperatively, and mate and breed within the group. Species of the genus have annual life cycles, but colonies of the social species may persist for several generations^57^. Besides cooperation in brood care and prey capture, social spiders cooperate in web building and nest maintenance. Solitary species reach on average 1.5–2 x larger adult body size than the social species (Fig. 1).

**Table 1:** Summary of the distribution and habitat of the six *Stegodyphus* species studied. References are in parentheses.

Species	Social/solitary	Distribution	Habitat
*S*. *africanus*	solitary	Central and Southern Africa	Arid and semiarid habitats; co-occurs with social species, *S*. *dumicola* or *S*. *mimosarum*^51,54^
*S*. *lineatus*	solitary	Widespread through the Mediterranean and North Africa, to Tajikistan	Dry or seasonal watercourses, with clustered distribution within habitats^79^
*S*. *pacificus*	solitary	India, Iran and Pakistan	Arid and semiarid habitats; co-occurs with social *S*. *sarasinorum*^51^
*S*. *dumicola*	social	Central and South Africa	Arid, grazed areas. Nests occur in shrubs and bushes, but also in tree tops in savanna areas^51^
*S*. *mimosarum*	social	Africa and Madagascar	Same arid habitats as *S*. *dumicola*, but often in trees near water^54^
*S*. *sarasinorum*	social	India, Sri Lanka, Afghanistan and Nepal	Semi-arid, grazed areas^80^

### Study design

To compare dietary niche width and breadth, we calculated dietary niche from the fraction of prey caught out of the amount of potential prey available at each individual nest. To do this, each nest was sampled simultaneously for potentially available prey in the immediate vicinity of the nest using various traps, and for actual prey captured by spider + web (or colony + web). *Stegodyphus* nests are stationary, and the spiders in their webs capture insects selectively from the immediate surroundings. Thus, in this paired design, variation in prey availability, assessed by trapping at the nest site, is directly comparable with the adjacent web captures. This design therefore incorporates the local variation in potential prey at the same small scale as the prey captured in an individual nest.

### Available and captured prey

Field studies on all six species were conducted in the period when subdadult or young adult females were present in the nest. This period is an important time for resource acquisition as fecundity is related to body size in females^48^. In total, we studied 14 different populations, seven each of solitary and social species (Table 2). We refer to a population as an assembly of solitary individuals or of social nests at a given location.

**Table 2:** Summary of experimental design in the field sites, showing the time period of sampling, geographic locations where we studied three social (*S*. *dumicola*, *S*. *mimosarum* and *S*. *sarasinorum*) and the solitary (*S*. *africanus*, *S*. *lineatus* and *S*. *pacificus*) species, number of nests used for observations and number of traps for insect sampling in each site (=population).

Species	Period (duration of trapping)	Site	Site name (coordinates E, N)	Number of traps	Number of nests	Total prey in traps	Total prey in webs
*S*. *africanus* 18.3 mm	South Africa Nov-Dec 2012 (35 days)	SA1	Witz (31.10, −24.55)	6	9	2830	37
		SA2	Hspr (30.95, −24.35)	7	4	5961	26
*S*. *lineatus* 16.3 mm	Israel April-May 2010 (17; 35; 35 and 21days)	Isr1	AF (34.78, 31.33)	8	37	1728	45
		Isr2	Leh (34.83, 31.36)	9	47	6053	155
		Isr3	ZA (34.78, 30.79)	9	14	6502	27
		Isr4	SII (34.73, 30.83)	8	12	2552	31
*S*. *pacificus* 17 mm	India Sep-Oct 2010 (24 days)	Ind1	Agastya (78.25, 12.88)	3	7	299	7
*S*. dumicola* 10.1 mm	Namibia Dec 2009- Jan 2010 (35 days)	Nmb1	H (17.23, −19.55)	9	11	3249	188
		Nmb2	HH (17.20, −19.48)	9	11	2689	95
		Nmb3	Usb (17.23, −19.55)	7	9	2802	82
*S*. dumicola* 10.1 mm	South Africa Nov-Dec 2012 (35 days)	SA1	Witz (31.10, −24.55)	3	3	1815	35
		SA2	Hspr (30.95, −24.35)	8	10	8166	160
*S*. mimosarum* 9.2 mm	South Africa Nov-Dec 2012 (35 days)	SA1	Witz (31.10, −24.55)	6	4	3840	38
*S*. sarasinorum* 10.2 mm	India Sep-Oct 2010 (24 days)	Ind1	Agastya (78.25, 12.88)	21	24	2323	166

Location coordinates are given in decimal degrees. Number of nests represent the number of individuals studied in each population of solitary species, and the number of colonies studied in the populations of social spiders. Total prey is the total number of insect prey observed in webs and traps for each population. Social species marked with*. Total body length is shown for each species.

To obtain data on the availability and utilization of prey by spiders in their natural habitat, we quantified the number of insect prey, insect size and the prey type defined by taxonomical order. Quantitative data on potential prey abundance and prey type were collected using one window trap and two sticky traps near every solitary or social nest selected for observations, placed at approximately the same height as the capture webs (Table 2). Window traps consisted of two Plexiglas sheets (2 mm thick, 40 × 30 cm) placed perpendicular to one another in the form of a cross, and situated on top of a funnel (31 cm diameter) attached to a collection bottle (1L) containing preservative liquid (20% ethylene-glycol (Namibia, India, South Africa) and 75% alcohol (Israel)). These window traps were designed to intercept flying and jumping prey^58^. Sticky traps, made of transparent 210 × 297 mm A4 plastic sheets with non-drying glue paste (Tree Tanglefoot (Tanglefoot company) (Namibia; India, South Africa) and Rimifoot (Rimi Ltd., Petach Tikva) (Israel)), were used to trap small flying insects. The combination of these two trapping methods was designed to catch available prey of a range of sizes and types. Trapping does not provide an absolute measure of prey availability, and therefore the term ‘available prey’ should be understood as potentially available prey trapped with these two sampling methods in the immediate vicinity of spider nests.

Concurrently, we recorded prey captured by spiders by observing their capture webs in the field during daytime, as previous studies showed that *Stegodyphus* capture most of their prey during the day^59^ (Y. Lubin unpublished). Each nest was observed eight times over three-hour periods on consecutive days, for a total of 24 hours in total, during which we noted the number and size of prey naturally captured by each individual or group. We treated each prey item captured by the individual or group within the observation period as an independent event. The surveys were done in consecutive days by the same two researchers across all the locations, and the size, taxon and placement of each prey item was carefully recorded to avoid double counting. Three social nests of *S*. *sarasinorum* in India were destroyed and disappeared during the study period, while the webs of eight nests were damaged by the heavy rains at the end of November. Our observation effort for this species was therefore 18–21 hours for 11 nests, and 24 hours for 4 nests.

The number of prey, prey size measured as body length in mm, and taxonomic order were recorded for every insect caught in webs or traps. Identification was done to order, rather than family or lower taxonomic level, due to the difficulty of identifying insects in the webs without disturbing the spiders. Body length of each prey item was measured with callipers to the nearest mm from the tip of the head to the end of the abdomen. We never removed prey items from the webs, and took care not to record a prey more than once by keeping track of prey size and taxon noted on the previous observation. Since *Stegodyphus* webs are two-dimensional and positioned vertically in relation to the ground, it was possible to measure the length of prey items without removing them. Subsequently we used a set of taxon-specific power equations to estimate dry biomass [mg] from prey length^60^. Prey length and mass, as well as wet mass and dry mass are highly correlated^18,60,61^.

### Group size estimates

Nests of social spiders collected in the study areas (N = 48 for *S*. *dumicola*, and N = 24 for *S*. *sarasinorum*), and in wooded savanna habitats across South Africa for *S*. *mimosarum*, (N = 23, data kindly provided by M Greve), were measured by length, width and height, and nest volume was estimated using the formula for the volume of two pyramids (volume of a pyramid: $\,V=\frac{1}{3}\times lwh$) as an approximation^48^. Each of these nests was opened to count the total number of spiders present. We used linear regressions to assess the relationship between nest volume and group size as a linear model provided the best predictor of group size among several approximations (*S*. *dumicola*, y = 0.2475x + 103.76, R² = 0.35; *S*. *mimosarum*, y = 0.1291x + 28.356, R² = 0.35; *S*. *sarasinorum*, y = 0.4068x + 47.671, R² = 0.69; Fig. S2 in Supplementary Analyses B). Subsequently, we used nest volume as a proxy for group size to avoid destruction of the natural nests involved in data collection.

Estimated group sizes ranged from 109 to 1450 individuals per nest in the Namibian populations of *S*. *dumicola*, and 115–256 individuals in South African ones. Nests of *S*. *mimosarum* in South Africa contained between 33 and 54 individuals, and Indian *S*. *sarasinorum* nests were 64 to 410 individuals per nest (all extrapolated from the nest volumes measured *in situ* and the regressions given above). Nests of *S*. *mimosarum* at SA1 site were smaller than those of the other two social species we studied (glmm coefficient effects t (*mimosarum*) = −2.22, p = 0.03).

Prey capture rate was calculated as the number of prey captured per capita per hour.

### Web size

For social species, capture web size was measured by estimating web area of a rectangle (*height* ∗ *width*). In case of more than one web sheet, each one was measured separately and summed to obtain the total web area. To obtain the web area per capita, total web area was divided by the estimated number of spiders in each nest of the relevant social species. Web size for solitary species was estimated by a rectangle if the web had four sides (*height* ∗ *width*) or a triangle if the web had three sides $(\frac{1}{2}\ast base\ast height)$.

### Data analysis

#### Dietary niche width

The range of insect prey sizes utilized (niche width) was estimated as the total niche width component of Roughgarden’s index^1^. Accordingly, total niche width (TNW) of a population can be broken down into two components: variation in resource use within individuals (or social group) (WIC), and the variance between individuals (or group) (BIC). It follows that total niche width equals TNW = WIC + BIC.

Estimates of TNW and WIC are based on a matrix of a resource measure, i.e. size (*x*_*ij*_) of *j*th prey item caught by an individual or group (*i*), for all the individuals (or social groups) in a population. The ratio of WIC to TNW, also known as Roughgarden’s index, measures the extent to which the total niche width of a population is due to individuals (or groups) within the population being resource generalists. The index is the ratio of WIC (within-individual component of size variance, WIC = E(Var(x_j_|i)) and TNW (total niche width, TNW = Var(x_ij_)):

*R’s I* = *WIC/TNW*. Values approaching one indicate that all individuals/groups utilize the full range of resources, whereas smaller values indicate decreasing inter-individual overlap and hence greater individual specialization. We included all the individuals of solitary species and colonies of social species that consumed more than two prey items, and weighted the estimations by the number of items consumed. Since none of the *S*. *pacificus* individuals we observed caught more than two prey items during our observation bouts, we did not calculate niche width measures for this species.

Monte Carlo simulation was implemented in the RInSp package^62^. We performed a nonparametric analysis that creates replicate, null diet matrices from the population distribution. These serve as null distributions and sampled with replacement to calculate p-values. The exact procedure was a follows: First, the exact number of prey captured by each individual (n_i_) in the population was determined. Subsequently, the resampling randomly reassigned each individual n_i_ prey item drawn from the population distribution of items. This was repeated 10000 times. The resampled population served as a null model corresponding to a population composed of generalists that sample randomly from a population’s diet, and have a sample size (number of prey items) equal to those of the observed data set.

TNW and WIC of populations of social vs. solitary species were compared using one-sided Mann-Whitney tests.

As an additional measure of prey utilization, we assessed the proportion of variance in prey size attributable to nests (webs) out of the total variance in captures by webs and traps combined. Variance of prey sizes was calculated for each nest and corresponding traps. The total variance was calculated by summing variances of webs and traps and the proportion of prey size variance in webs out of the total variance was then computed for each nest. The proportions were analyzed in relation to the foraging mode (group or solitary) and site. We arcsin transformed proportional variance as a response variable, and fitted a generalized linear mixed model with Gaussian error distribution, using foraging mode as an independent variable, and sampling method (web/trap) nested within site as a random effect. ANOVA statistics in the form of likelihood-ratio chi-squares are reported.

The population niche width and indices of individual/group specialization on prey size were calculated using the RInSp R package^62^.

#### Dietary niche breadth

Measures of individual and population level niche breadth, respectively, were derived as follows: A likelihood measure of diet breadth, the Petraitis index^63^, was derived for each nest of solitary and social species, where *λ*_*i*_ is the likelihood ratio of the observed resource use of a given nest against the population resource distributions (total niche breadth, calculated from pooled trap and web captures), and D_*i*_ is the number of diet items recorded in the diet of that individual (*i*). For a complete generalist, W_*i*_ = 1, and the value decreases with greater specialization. The likelihood of the observed diet of individual *i* is: λ_i_ = ∏j(q_j_p_ij_)n_ij_, where q_j_ is the population proportion of the resource *j*, p_ij_ is the proportion of the resource *j* in the diet of the individual *i*, and n_ij_ is the number of items for individual *i* and resource *j*. This can be used to calculate a p-value to test the significance of the diet specialization using the generalized likelihood ratio test.

We calculated the proportion of variance in prey type attributable to nests (webs) out of the total captures by webs and traps combined, using a modification of Petraitis’ index of niche breadth. Counts of different prey types were computed for each nest and trap by pooling the two samples together. Based on these combined counts we calculated niche breadth for a population of a given species at a given site. The same was done for web samples only, so that the niche breadth was calculated for a population based on web captures of nests of a given species at a given site. The proportion of niche breadth of webs out of the total captures by webs and traps combined was calculated by dividing the niche breadth in webs by the total niche breadth.

We examined whether there was taxon-specific prey size selection by comparing prey sizes of each insect order found in *Stegodyphus* webs with prey sizes in their surrounding habitat (traps). Possible size differences of prey captured in traps and in webs were compared using penalized quasi-likelihood generalized linear mixed models (GLMM), with quasi-Poisson family specification to correct for overdispersion. The models included interaction effects of the capture method and prey order.

### Individual biomass intake and capture web size of social and solitary foragers

Mean individual biomass intake rate of social and solitary species was compared by fitting a generalized linear mixed model with Gamma error distribution and log-link, using cooperative or solitary foraging mode an independent variable, and species nested within location as a random effect. This was done with the glmer function from the lme4 package in R. Individual web production in relation to foraging mode was analysed in a similar way. Individual biomass intake was divided by individual web production to obtain an estimate of biomass intake relative to web production per capita. We ln-transformed this ratio as a response variable, and fitted a generalized linear mixed model with Gaussian error distribution, using foraging mode as an independent variable, and species nested within location as a random effect. ANOVA statistics in the form of likelihood-ratio chi-squares are presented.

### Group size effects

The effect of group size on the size of capture webs was analysed by generalized linear mixed models fit by REML, using lme4 package for R. Nest size and site were specified as fixed effects, and species nested within site as a random effect. We retained nest size as the single independent variable after backwards elimination of non-significant factors in the initial model. Data were log or double square-root transformed where necessary to satisfy assumptions of normality.

The effect of group size on prey size was tested by using a generalized linear model with quasipoisson distribution for the response. Group size, prey order and their interaction were set as fixed, and nest ID nested within site as a random effect.

Mean prey biomass per capita consumed per nest, and total prey number caught per hour by groups of the three social species were compared by generalized linear mixed models fitted by REML, using lme4 package for R. Nest size and site were specified as fixed effects, and species nested within site as a random effect. We dropped the random effect by backwards elimination of non-significant factors in the initial model. Data were log or double square root transformed where necessary to satisfy assumptions of normality.

The effect of group size on variance in per capita biomass intake, calculated as the standard deviation of biomass of all the prey captured by each social nest and specified as Gamma family distribution, was analysed with glmm, specifying nest volume as independent variable and species nested within location as random effect.

Variance inflation factors of the models were checked using the vif function in Car package for R. All statistical analyses were performed in R version 3.3.2. The data and analyses are available from the corresponding author upon reasonable request.

## Results

### Dietary niche width

Our data show that the social species foraged on a broader range of prey sizes and therefore had a wider dietary niche than that of solitary species (Fig. 1 and ***TNW web*** in Table 3). Within-individual niche variation (i.e. the variation in prey sizes captured by each social and solitary nest) was higher in social compared to solitary species (***WIC*** in Table 3) and the proportional variance as well was higher for social species (Table 3). The variance of foraging mode nested within site as the random effect in the model was 0.128. The available prey collected from traps in the vicinity of each observed nest showed similar prey size spectrum and did not differ significantly between micro-habitats occupied by nests of social and solitary spiders (***TNW environment***, Table 3; and additional results in the Supplementary analyses A).

**Table 3:** Summary statistics of indices of foraging niche width and breadth for *Stegodyphus* spiders.

Species	Site	Niche Width				Proportional variance	Niche Breadth	
		TNW *environment*	TNW *web*	WIC	Individual specialization (R’s I)		W_*i*_	Proportional variance
*S*. *africanus*	SA1	63.055	35.580	27.654	0.777 (n.s.)	0.758 ± 0.121	0.399 ± 0.134	0.650
	SA2	47.03	13.175	12	0.911 (n.s.)	0.705 ± 0.292	0.540 ± 0.215	0.732
*S*. *lineatus*	Isr1	21.559	10.25	2.865	0.280 (0.02)	0.406 ± 0.217	0.338 ± 0. 177	0.676
	Isr2	62.482	12.941	4.800	0.371 (0.002)	0.365 ± 0.171	0.454 ± 0.237	0.726
	Isr3	30.126	14.777	8.662	0.586 (n.s.)	0.357 ± 0.269	0.472 ± 0. 236	0.899
	Isr4	24.887	10.21	8.007	0.784 (n.s.)	0.589 ± 0.180	0.398 ± 0.156	0.655
*S*. *pacificus*	Ind1	NA	NA	NA	NA	0.345	NA	NA
*S*. dumicola*	Nmb1	70.213	44.649	43.105	0.965 (n.s.)	0.747 ± 0.053	0.793 ± 0.147	0.933
	Nmb2	81.505	79.844	62. 957	0.789 (n.s.)	0.742 ± 0.123	0.556 ± 0.213	0.737
	Nmb3	44.612	37.187	25.401	0.683 (0.004)	0.617 ± 0.207	0.601 ± 0.150	0.785
	SA1	12.174	31.093	20.576	0.662 (0.005)	0.607 ± 0.237	0.762 ± 0.105	0.985
	SA2	49.154	53.796	49.410	0.919 (n.s.)	0.777 ± 0.141	0.538 ± 0.223	0.756
*S*. mimosarum*	SA1	30.371	21.356	17.848	0.836 (n.s.)	0.531 ± 0.344	0.760 ± 0.194	0.789
*S*. sarasinorum*	Ind1	47.696	48.157	35.927	0.746 (n.s.)	0.608 ± 0.246	0.438 ± 0.242	0.608
ANOVA statistics *SOCIAL VS*. *SOLITARY*		n.s.	W = 2 (0.002)	W = 3 (0.004)	—	χ^2^ = 5.74 (0.002)	χ^2^ = 15.94 (<0.0001)	n.s.

Measures of niche width are based on prey size: total niche width of potential prey (TNW environment), total niche width of spider prey (TNW web), within-individual (nest) component of size specialization (WIC), individual specialization for prey size (R’s I, bootstrapped p-value), and proportion of variance of prey size in webs out of total variance are shown. The likelihood measure of niche breadth based on prey type (Petraitis W*i*, mean ± SD) is shown, and the proportion of variance of prey type in webs out of the total variance. Significant differences between population niche estimates of social (*S*. *dumicola*, *S*. *mimosarum* and *S*. *sarasinorum*) vs. solitary species (*S*. *africanus*, and *S*. *lineatus*) are shown in the last row (Type II Wald χ^2^ test of sociality in glmm; one-sided Mann-Whitney tests where parametric tests were not applicable). Social species marked with*.

We found limited evidence of individual specialization for prey size (***R***’***s I***, Table 3). Bootstrapped values of individual specialization within different populations in most cases ranged from 0.7–1 (with 1 indicating a generalist diet). Among solitary species, however, two populations of *S*. *lineatus* (sites Isr1, Isr2) showed significant specialization as indicated by low *R*’*s I* index. In these populations, spiders caught smaller sized prey, resulting in narrower niche width. Specializing on smaller prey resulted in reduced within-individual variance components of the niche indices in *S*. *lineatus* (Table 3). Among social species, two populations of *S*. *dumicola* also showed some specialization (sites Nmb3 and SA1, Table 3), with total niche width and within-individual variation in prey size for both of these populations being at the lower end of the range of values recorded among the social spiders.

A separate analysis of niche width using only nests of social and solitary species that co-occurred at a site yielded no significant difference between solitary and social species (Fig. S1a in Supplementary analyses A). Low sample sizes for solitary nests (*S*. *africanus*) at SA2 and solitary nests (*S*. *pacificus*) at Ind1 (see Table 1 for sample sizes), as well as the omission in the analysis of *S*. *dumicola* at the sites in Namibia and *S*. *lineatus* in Israel, may explain the discrepancy (further discussion in Supplementary analyses A).

### Dietary niche breadth

**Figure 2:**
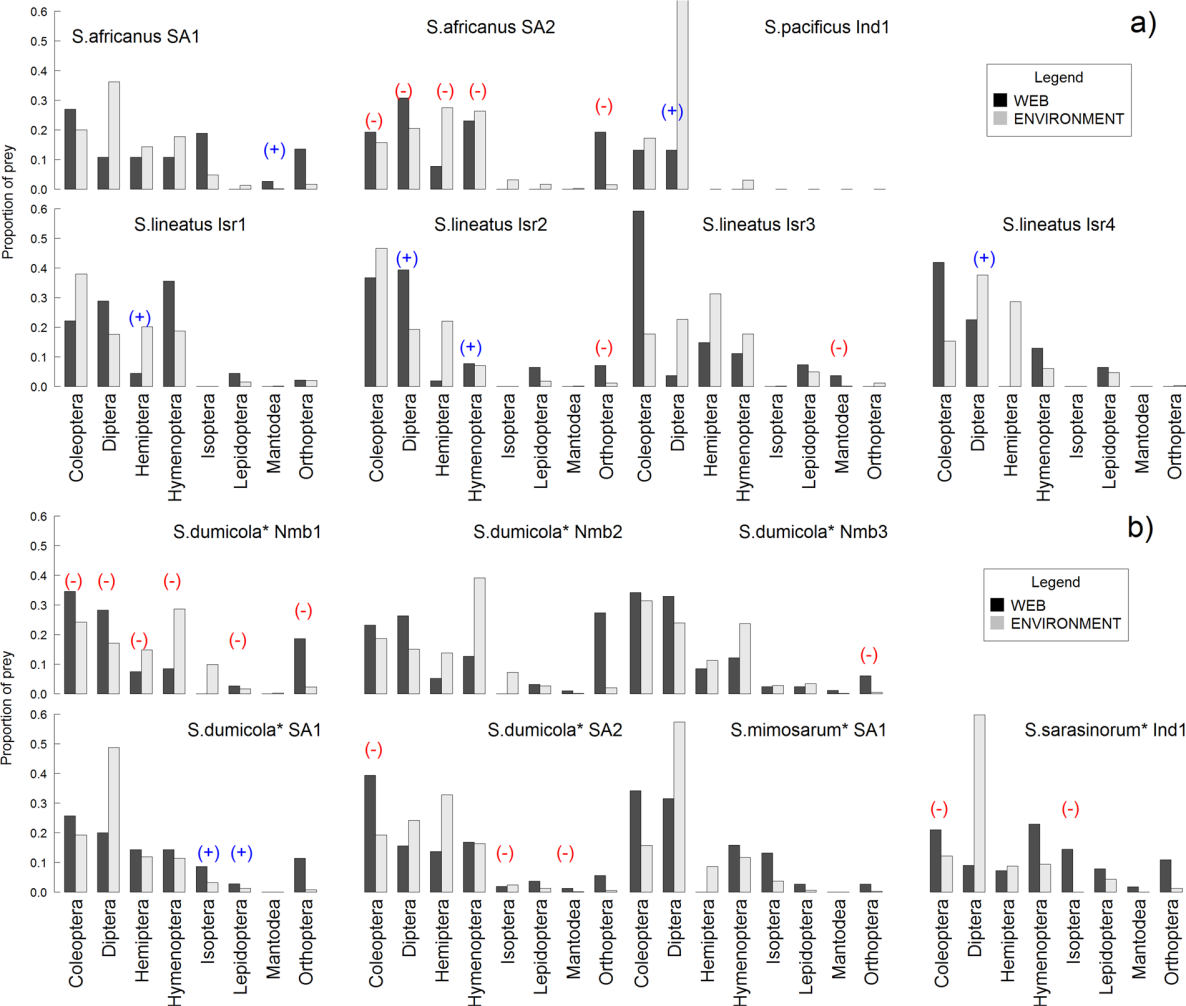
Percentages of the most frequent prey taxa caught in the webs and traps in each site: (**a**) Solitary species and (**b**) social species of *Stegodyphus*. Black bars represent their frequency in webs, while grey bars represent their frequencies in traps. Plus symbols within brackets (+) above the bars indicate that the respective prey order was of significantly larger size in the webs than in traps; minus symbol (−) indicates the opposite (rates of change in size were estimated from the exponents of coefficient estimates in the models; full analysis presented in Table S1). For details on the statistical analyses see Supplementary analyses A.

Social foragers showed an expanded dietary niche by including a broader range of taxonomic prey orders in their diets compared with solitary foragers (Fig. 2 and ***Wi*** in Table 3). Furthermore, likelihood estimates of niche breadth (***Wi web***) classified social spiders as more generalist (*Wi* approaches 1 for a complete generalist) than solitary foragers (Table 3, glmm with sociality as fixed effect and species nested within site as random effect; sociality effect χ^2^ = 15.94, p < 0.0001). *Wi* values showed large standard deviations (Table 3), and within species there was significant variation in dietary niche breadth among different locations (random effect χ2 = 13.5, df = 1, p < 0.0001, colony nested within location random effect variance 0.04). All of the species used a large proportion of the prey types available from the overall sample of webs and traps combined. Social species tended to use a larger proportion of prey types from the total samples than the solitary ones, but the results were not statistically significant (proportional variance, Table 3). Analyses of the taxonomic range of available prey from traps in the vicinity of each observed nest revealed significant difference in the composition of prey taxa in micro-habitats of social vs. solitary species (Table S3 in the Supplementary analyses A).

Differences in nest height did not contribute to the difference in abundance of captured prey types. Nests of social species were generally higher in the vegetation than those of solitary species (Wilcoxon rank sum test with continuity correction, W = 30524, p < 0.0001), but the abundances of different prey orders trapped in spider webs were not correlated with nest height (Table S3 in the Supplementary analyses A).

### Opportunistic foraging or shift in resource use

We explored whether the increase in dietary niche of social species reflects opportunistic foraging on available insect prey, or indicates a shift in resource use to specialise on larger prey or different prey taxa. Similar taxonomic orders were found in habitats (available prey) and webs (captured prey) across study sites (Fig. 2). No taxonomic order was captured exclusively in the webs of social species, therefore there was no evidence for a shift in prey type use, although the frequency of capture of different orders differed among social and solitary foragers (Fig. 2 and Table S2 in the Supplementary analyses A). The most abundant prey taxa trapped in the environment were Coleoptera, Diptera and Hymenoptera. Spiders also caught other, less abundant prey taxa such as Isoptera, Lepidoptera, and Orthoptera (Fig. 2 and Table S2 in the Supplementary analyses A), however large orthopterans in particular may have been under-sampled in the traps due to their ability to escape.

For each prey order, we compared insect size distributions between webs and traps, and although some species caught larger sized prey in the webs relative to the available prey sizes, we found little consistent evidence for size-specific specialization within each prey taxon (Fig. 2). This result held also for two of the three sites where solitary and social species overlapped (Table 3 and Fig. S1 in the Supplementary analyses A). Our data collectively suggest that social species expand dietary niche by opportunistic foraging rather than by specialization on large insects or specific prey types.

### Individual biomass intake and capture web size of social and solitary foragers

We calculated individual consumption rate (biomass intake in mg per capita over the 24 h observation period) for social and solitary species (Table 4). Solitary spiders obtained approximately 100 times higher per capita biomass than individuals of social species (glmm solitary species coefficient estimate t = 9.122, p < 0.0001; sociality effect χ2 = 83.216, df = 1, p < 0.0001; random effect variance of species nested within site in the model = 0.017). We also calculated per capita capture web production in social and solitary species, and found that social spiders on average produced in the range of 3–22 fold less capture web per individual than solitary species, suggesting that cooperative trap building confers energetic benefits in terms of reduced silk production (Table 4; glmm solitary species coefficient estimate t = 14.11, p < 0.0001; sociality effect χ^2^ = 198.98, df = 1, p < 0.0001; random effect variance of species nested within site in the model = 0.003). Combining these two metrics, the mg prey biomass obtained per unit capture web per individual was significantly lower in social compared with solitary species (glmm solitary species coefficient estimate t = 4.176, p < 0.0001; sociality effect χ^2^ = 17.436, df = 1, p < 0.0001; random effect variance of species nested within site in the model = 0.248). Therefore, despite an energetic benefit in terms of reduced individual silk production, cooperation did not overall provide higher biomass intake.

**Table 4:** Per capita estimates of prey biomass, web size scaled to nest size (web area divided by nest volume), and their ratio, and the range of group sizes of all species studied.

Species	Prey biomass (mg ∗ cm^−3^) (mean ± SE)	Web size (cm^−1^) (mean ± SE)	Prey biomass/Web size (mg ∗ cm^2^) (mean ± SE)	Group size (range)
*S*. *africanus*	21.138 ± 4.785	574.900 ± 157.757	0.118 ± 0.065	1
*S*. *lineatus*	6.597 ± 1.058	339.323 ± 41.993	0.097 ± 0.032	1
*S*. *pacificus*	21.759 ± 9.692	492.071 ± 140.372	0.044 ± 0.020	1
*S*. dumicola* (site NMB1-3)	0.161 ± 0.075	23.479 ± 3.190	0.010 ± 0.005	109–1450
*S*. dumicola* (site SA1, SA2)	0.097 ± 0.031	21.450 ± 4.574	0.006 ± 0.002	115–256
*S*. mimosarum*	0.114 ± 0.058	26.792 ± 11.647	0.007 ± 0.003	33–54
*S*. sarasinorum*	0.185 ± 0.037	15.539 ± 2.954	0.021 ± 0.007	64–410

Prey per capita was estimated from mean prey biomass obtained by each nest through an observation period totalling 24 hours (for most nests, see Methods), which was divided by group size (for details on group size estimates of social species see the Methods). Web size for *S*. *pacificus* was taken from unpublished work based on data from the same period and area (L. Grinsted, pers. comm.). Social species marked with*.

Social spiders are smaller than solitary species. When correcting for the size difference by dividing per capita biomass by average body size of each species (from Fig. 1), prey per capita estimates were on average 15 times higher for solitary than for social species (sociality effect χ2 = 31.291, df = 1, p < 0.0001; random effect variance of species nested within site in the model = 7.985 ∗ 10^−7^).

### Group size effects

**Figure 3:**
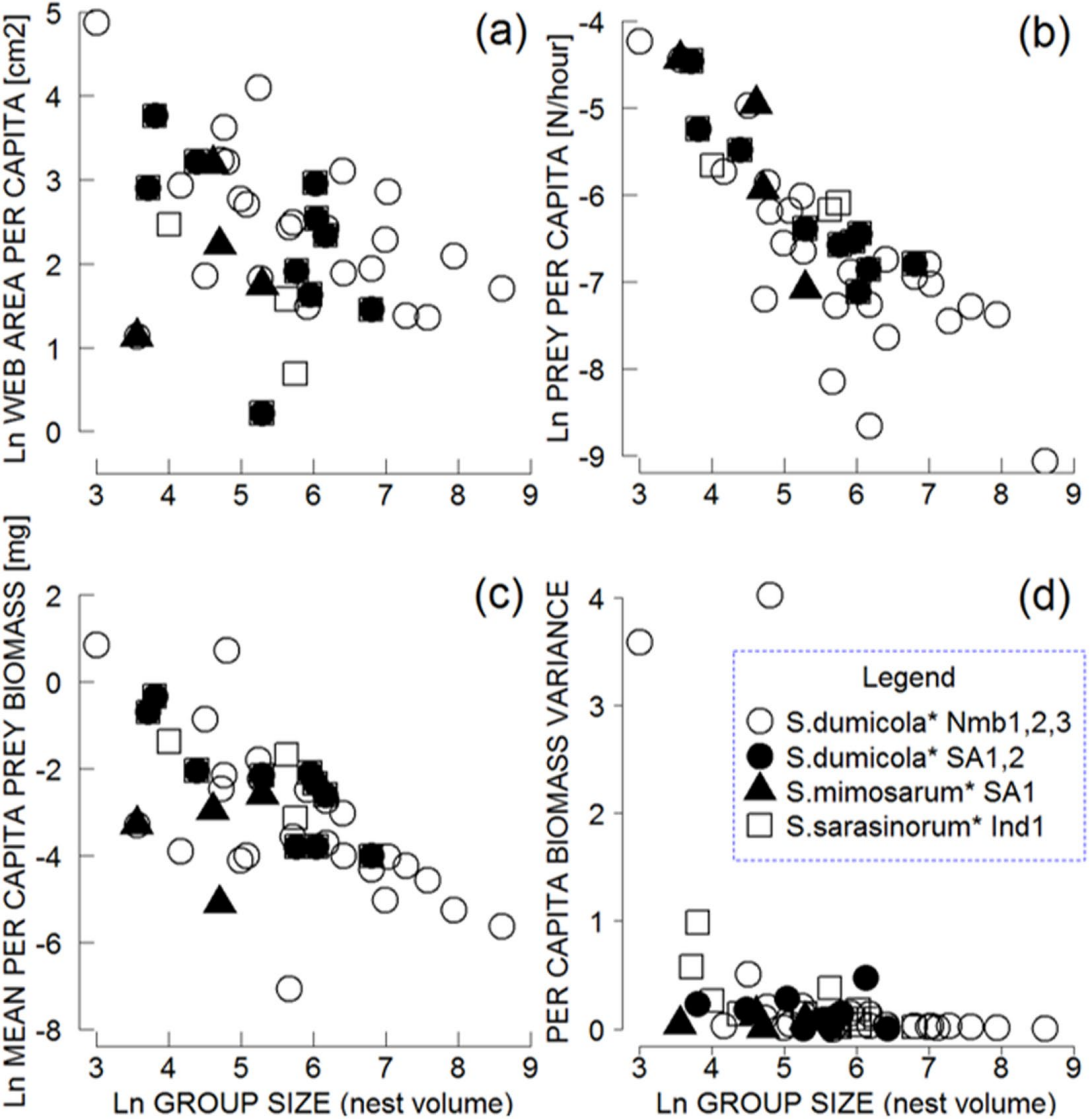
Web area per capita (**a**) prey capture rate per capita (**b**) prey biomass per capita (**c**) and per capita variance in biomass (**d**) in relation to group size for each observed nest of three cooperatively foraging species: *S*. *dumicola* (N nests = 25 in Namibia, 10 in South Africa, filled and empty circles, respectively), *S*. *mimosarum* (N nests = 4, filled triangles) and *S*. *sarasinorum* (N nests = 14, empty squares). Prey biomass is shown in mg; web area in cm^2^. We used nest volume as a proxy for group size, as they are positively correlated (see Methods section).

Within the social species, web area increased allometrically with group size (group size estimated as nest volume: mixed models group size effect t = 6.338; p < 0.0001; random effect of species nested within location χ2 = 10.5, df = 1, p = 0.001). Per capita capture web area decreased with increasing group size, suggesting that individuals in larger groups contributed relatively less silk to capture webs (Fig. 3a; mixed models group size effect t = −4.087; p = 0.0002; random effect of species nested within location χ2 = 10.5, df = 1, p = 0.001, variance = 0.111). Prey capture rates of social species increased with web size (generalized linear models web area effect t = 4.158, p = 0.0001; with a significant site effect χ2 = 21.968, df = 5, p = 0.0005; a weaker effect was found in one *S*. *dumicola* population NMB3; t = −2.288, p = 0.03). However, there was a diminishing rate of prey capture (number of prey/hour) with increasing group size (Fig. 3b; generalized linear models, nest size effect t = −2.753, p = 0.0009). This analysis showed a significant interaction term between group size and site (χ^2^ = 24.454, df = 5, p = 0.0002), suggesting that prey capture rate decreased more slowly in nests of *S*. *dumicola* at NMB1 and NMB3 sites, while the decrease was steeper in nests of *S*. *mimosarum* and *S*. *dumicola* at SA1 site. This effect was due to a narrower range of nest sizes and capture webs of the latter two populations.

Group size did not have any effect on prey sizes caught by social species (F_1,38_ = 0.2084; p = 0.65). The effect of prey order on prey size was significant (F_10,448_ = 20.431, p < 0.001), but there was no interaction between group size and prey order. Mean prey biomass obtained per capita, however, decreased with group size (Fig. 3c; generalized linear model, group size effect t = −3.121, p = 0.003). There was a significant interaction term of group size and location (χ^2^ = 28.543, df = 5, p < 0.0001), as per capita biomass decreased less with group size in *S*. *dumicola* at NMB3 site (t = 2.875, p = 0.006). Overall, prey-capture rate and estimated individual biomass intake declined with increasing group size (Fig. 3).

Finally, we assessed the potential for reduced intra-group variance in prey consumption (Fig. 3d). Variance in per capita biomass intake decreased with increasing group size (Wald chi square test of group size effect, χ2 = 28.49, df = 1, p < 0.0001; random effect of species nested within location variation = 0.19).

## Discussion

Group living animals are faced with the challenge of acquiring sufficient resources to meet the requirements of maintenance and reproduction, thus allowing the group to persist. Cooperative foraging strategies may provide a solution by expanding the dietary niche or facilitating exploitation of resources that are unavailable to solitary foragers. We used a comparative approach to investigate whether cooperative foraging allows social *Stegodyphus* spiders to expand or change their dietary niche. We compared the dietary niche of three social and three solitary species, based on the range of prey captured by spiders in their webs relative to prey availability in their immediate habitat. We found that there was no difference in the sizes of trapped insects (potential prey) in the micro-habitats of social and solitary spiders. Yet, the social species enlarged their dietary niche relative to solitary congeners by including large prey relative to the average size of potential prey recorded in the adjacent micro-habitat, thereby expanding dietary niche width, and also by including a wider taxonomic range of prey and thus broadening the dietary niche. These results support the hypothesis that cooperative foraging facilitates capture of larger prey sizes and a wider variety of prey types in social *Stegodyphus*.

As social and solitary *Stegodyphus* overlap partially in their distribution ranges^52^, theory predicts the evolution of differentiated foraging strategies within the ecological niche^2^, either by expanding the dietary niche or facilitating a shift in resource use. Our analyses show that social *Stegodyphus* species forage opportunistically in relation to available prey rather than selectively specializing on larger or specific prey types (Table 3). Thus, social species enlarged their foraging niche relative to solitary species, but they did not shift to a new niche. Even at sites where social and solitary species occurred in the same habitat, social *Stegodyphus* species were not more specialized than solitary congeners. Thus, the broader foraging niche of the social species appears to overlap and include within it that of the solitary species. Opportunistic foraging on both large and small prey in social *Stegodyphus* may also reflect a low relative abundance of large prey in the sub-tropical semi-arid grasslands where these species occur. Thus, while they are able to handle large insects by cooperating in prey capture, spiders in colonies do not ignore small insects trapped in their webs. These results contrast markedly with the New World *Anelosimus*. Social and solitary *Anelosimus* are largely segregated by elevation: social species occupy lower elevation sites and feed on larger prey that are scarce at higher elevations^20^. Where social and solitary species overlap, the social *Anelosimus* captured on average significantly larger prey relative to their less social counterparts^14,41^.

Geographic distribution and different life-history patterns may explain the distinct foraging niche responses of these two genera. Social and solitary *Stegodyphus* occur across Africa and Asia, with several species overlapping geographically; however, there is little elevational gradient and no separation between social and solitary species by elevation^52^. On a large-scale, geographic range, the social species in both genera occur in habitats characterized by higher productivity, and consequently high insect abundance, in comparison with habitats of their solitary congeners^15,37^. In *Anelosimus*, these habitats are tropical with relatively little climatic seasonality. The lack of strong seasonality in insect abundance enables *Anelosimus* colonies to remain active year-round, thus increasing the potential for competition with co-occurring solitary species. Elevational separation and foraging niche specialization may enable *Anelosimus* to overcome competition.

By contrast, both social and solitary *Stegodyphus* species typically occur in subtropical, semi-arid regions with strong precipitation seasonality, and consequently face a dry or cold season with low insect activity. During this period, *Stegodyphus* colonies and solitary species undergo a form of hibernation, reducing foraging activity and ceasing to maintain a capture web (^54,59^; YL personal observation). Reduced foraging activity corresponds to the period of maternal care, when newly emerged young are present in the nest. Females in the nest feed the young with regurgitated liquid stored in the digestive system, and the young eventually kill and consume the adults^34,54,64^, emerging to renew the colony capture web when insect abundance increases in spring. Climate and insect availability impose a strongly seasonal activity pattern and developmental synchrony on social *Stegodyphus* species^54,59,65^. Thus, both intraspecific and inter-specific competition may be avoided during the time of year when prey abundance is low. We suggest that reduced competition in *Stegodyphus* allows social and solitary species to overlap in their distributional ranges. Finally, additional mechanisms (e.g., differential susceptibility to predators, micro-habitat preferences) might enable overlap and coexistence of social and solitary *Stegodyphus* species.

Cooperative foraging is expected to yield energetic benefits to group members through greater prey capture efficiency and reduced variance in resource acquisition^9,66^. We examined whether an increase in the dietary niche of cooperatively foraging species also translated into higher per capita biomass intake, but found that on average, individual biomass intake of the social species was significantly lower than that of solitary foragers (Table 4). Individuals of social species, however, contributed substantially less to capture web production, since per capita unit web area was smaller in social compared with solitary species. Social species thereby gain energetic benefits from cooperative foraging, as silk is costly to produce^53^. Combining these two metrics, we found that individual prey intake obtained per unit capture web was still lower in social compared with solitary species. This result also holds when correcting for size differences between social and solitary species. Therefore, although gaining an individual energetic benefit by reduced capture web production, cooperative foraging did not provide social species with a higher individual biomass intake. We did not take into account costs of web renewal, which might be higher for solitary species with webs lower in the vegetation and therefore more prone to disturbance. If these costs are substantial, the balance might be shifted towards relatively greater energetic benefit to social species over solitary. This remains to be investigated.

As is the case in other social animals^9,67^, several lines of evidence suggest that competition for resources within social spider colonies increases with group size. We showed that prey capture rate and estimated individual biomass intake declined with increasing group size (Fig. 3). Similar patterns were reported in the social spider *Agelena consociata* from the equatorial African rainforests^31^, in *A*. *eximius*^13^, and in *Anelosimus guacamayos* (E. Yip pers. communication). In social *Stegodyphus* species, female body size decreased with increasing nest size^53,54,68^, and fecundity declined with increasing group size^48^. These observations support the hypothesis that the increase in competition with increasing group size negatively affects colony growth. The negative effects of group size, however, may be balanced by increased cooperation within groups. Feeding efficiency was greater in groups of the social *S*. *dumicola* than in single spiders^69^, and an experimental study showed that when food was limited, cooperative foraging allowed group members to exploit the available prey more efficiently^70^. Nevertheless, the food-limited spiders had a lower body mass.

Colony size may have important non-trophic benefits that counteract the increased cost of foraging. Bilde *et al*.^48^ found that the survival of *S*. *dumicola* nests increased with group size, and larger groups were better able to defend the nest against predators and parasites, for example predatory ant raids^71,72^. In *Anelosimus*, solitary individuals and small colonies suffered high predation rates, in particular by ants, in the lowland tropical rain forest, a factor that could favour group living^38,73–75^. These findings suggest that improved predator defence with increasing group size is an important factor in the maintenance of group living in spiders.

Food shortage and interference competition over prey during communal feeding results in asymmetric rewards for cooperatively foraging individuals^55,76^. Under a scenario of contest competition^50,77^, some individuals may not succeed in obtaining enough resources to breed^42,43,70^. In social spiders, allo-maternal care and smaller clutch sizes may have evolved to mitigate these effects of resource competition and resulting reproductive skew^64,70,78^. Cooperative foraging may also reduce the variance in prey capture and individual consumption rate, which buffers the group against starvation and increases the chance of successful reproduction^12,28–30^. Our analysis showed that variance in individual biomass intake decreased with increasing group size (Fig. 3d), an effect that could represent an important benefit of group living in spiders. Although per capita rate of biomass uptake is low in large groups, smaller groups that experience a higher variance may drop below the threshold for survival. Indeed, small nests of social spiders frequently experience high mortality rates^46,48^.

In conclusion, our study provides comparative evidence for the hypothesis that cooperative foraging increases dietary niche in social spiders through opportunistic foraging. Social *Stegodyphus* species expand dietary niche width by including prey of relatively larger size, and dietary breadth by including a broader taxonomic range of prey types in the diet, compared with solitary congeners. This implies that opportunistic foraging rather than resource specialization facilitates co-existence with solitary congeners. The social *Stegodyphus* species acquire energetic benefits from reduced individual capture web production, however, per capita prey capture rate and biomass intake decrease with increasing group size, indicating that cooperation does not offset costs of competition. Given that social groups are sedentary and dependent on the stochastic arrival of insect prey in their capture webs, a generalist and opportunistic foraging strategy may be the only way cooperative foragers can meet energetic demands. Larger groups experienced reduced variance in individual intake rate, indicating an important benefit of group living that contributes to their persistence.

## Electronic supplementary material

Supplementary Analyses
