# Supplementary material for: Cooperative foraging expands dietary niche but does not offset intra-group competition for resources in social spiders
Source: Sci Rep. 2018 Aug 7;8:11828. doi: 10.1038/s41598-018-30199-x (PMC6081395; doi:10.1038/s41598-018-30199-x)
Supplement: Supplementary file 1 — Supplementary Analyses [file 41598_2018_30199_MOESM1_ESM.pdf]

Supplementary material_ ***Cooperative foraging expands dietary niche but does not offset intra-group competition for resources in social spiders.*** Authors: Marija Majer, Christina Holm, Yael Lubin & Trine Bilde

SUPPLEMENTARY ANALYSES: A. COMPARISONS OF PREY SIZE IN TRAPS VS. WEBS

Methods:

For each species, prey sizes captured in the paired traps and webs were compared by penalized quasi-likelihood generalized linear mixed models (GLMM), using the glmmADMB package for R. We used the quasi-poisson family specification to correct for overdispersion. We specified capture method (trap/web), site and prey type as covariates, and set trap/web ID as a random effect. To avoid singularity error when running models, we excluded all spider specimens caught in traps or webs as their counts were very small across the data.

The size of prey was compared across all the species with penalized quasi-likelihood generalized linear mixed models, first for the insects caught in traps, and then for the prey recorded in the webs. Here we specified sociality, site and in case of traps, trap type, as covariates, and set trap/web ID and prey type as random effects. In order to check assumptions of GLMM, model residuals were inspected graphically. Prey type (order) was set as a random effect, since we investigated potential effects on prey size selection in these analyses. See analysis of specific prey taxa differences in the main text.

Additionally, we analysed resource use in relation to the abundance of different prey orders in the corresponding habitat, first by computing F tests on contingency tables of the respective prey types. To test whether more abundant insect types were also preyed upon more often (selectivity based on environmental abundance, i.e. expected count), we calculated the standardised residuals for each prey type (*SR= (observed count-expected count)/square root (expected count)*). Correlation of SR to the frequency of corresponding prey types in the environment (i.e., the expected value of their consumption rate) was inspected for each population.

Supplementary material_ ***Cooperative foraging expands dietary niche but does not offset intra-group competition for resources in social spiders.*** Authors: Marija Majer, Christina Holm, Yael Lubin & Trine Bilde

In order to compare abundance of different insect orders in webs, sticky and window traps, we computed Chao and Morisita-Horn similarity indices. Chao's index is an abundance-based similarity index that takes into account the effects of unseen species (Chao *et al.* 2005), while Morisita-Horn is another abundance-based similarity index derived from the Sørensen index that is less sensitive to differences in species richness and sample size (Wolda 1981). We further used permutation manova method (Anderson 2001) to assess the difference in abundances of different insect orders in relation to location, nest/trap height and species.

Results:

We examined the range of prey sizes and types (i.e. taxonomic orders) in *Stegodyphus* webs vs. trap captures in their vicinity. Both solitary and social spider species caught on average larger prey in their webs than the insects caught in traps (Fig.1; ANOVA results for each species individually in Table S1). For the two social species, *S. mimosarum* and *S. dumicola* studied in site SA1, there was no significant difference in prey size between traps and webs (Fig. 1, Table S1). Prey order, collection date, and their interaction term were significant for all of the sites except for Isr3 and Isr4, where we studied the solitary *S. lineatus.* A significant interaction term between prey order and collection date indicated that different prey types differed in size depending on the collection date. The largest insects caught in webs and traps (taking the 75% quartile of body length) belonged to the insect orders Orthoptera, Mantodea, Isoptera and Lepidoptera.

<u>Prey size in traps (environment)</u>

The sizes of prey caught in traps did not differ between traps associated with nests of solitary or social species (TNW *environment*, Table 3). There was a significant effect of trapping method on the size of prey caught, with window traps catching larger prey ($t= 73.03$, $p<.0001$). The only significant coefficient estimate indicating site effects was for Isr4, where insects caught in the traps were smaller than in the rest of the sites ($t= -2.44$, $p= 0.02$). There was considerable variation in the sizes of different prey types caught by each trap, as the variance of prey type nested within trap ID in the model was 0.54. No differences in prey size in the traps were detected between co-occurring species (non-significant comparisons: solitary *S. africanus* vs. social

S. dumicola and *S. mimosarum* in SA1; solitary *S. africanus* vs. social *S. dumicola* in SA2; solitary *S. pacificus* vs. social *S. sarasinorum* in Ind1).

Prey size in webs

There were location effects on the sizes of prey caught in webs, whereby two of the four populations of solitary *S. lineatus* caught prey of smaller size range than the remaining species (Isr1 t=-2.12, p=0.04; Isr2 t=-2.07, p=0.04), while the size range of prey caught in the webs at site Ind1 was larger than in the rest of our samples (t= 2.71, p=0.008). Both the social *S. sarasinorum* and solitary *S. pacificus* we studied at this site caught relatively larger sized prey, with median sizes above the rest of the population samples (see Fig.1). Variance of the prey type nested within nest ID in the model was 0.403.

Comparing the prey size caught in the webs of co-occurring species, we detected a significant difference between species co-occurring only at site SA1. At this site, solitary *S. africanus* caught significantly larger prey than cooperative *S. dumicola* and *S. mimosarum* (Fig.S1 a); t= 3.09, p=0.01). This is also reflected in the higher values of niche estimates for *S. africanus* at the respective site (see Table 3), and is consistent with a lack of statistically significant differences in prey size caught in traps and webs of the two social foragers in the same site (Table S1; sample size was three for *S. dumicola*, and four for *S. mimosarum*, respectively).

Prey abundance

Abundances of prey types caught by the spiders in their webs differed from those caught in the traps (Table S2). Abundances of different prey orders caught in the webs varied among species but not among study sites nor in relation to nest height (Table S3). Abundances of different prey orders caught by sticky traps differed strongly among species and location (comparisons based on Chao and Morisita-Horn similarity indices, Table S3). The same results were obtained when comparing prey abundances in sticky traps and window traps (Table S3), where the abundances of different prey orders were independent of trap height for both trap types.

Supplementary material_ ***Cooperative foraging expands dietary niche but does not offset intra-group competition for resources in social spiders.*** Authors: Marija Majer, Christina Holm, Yael Lubin & Trine Bilde

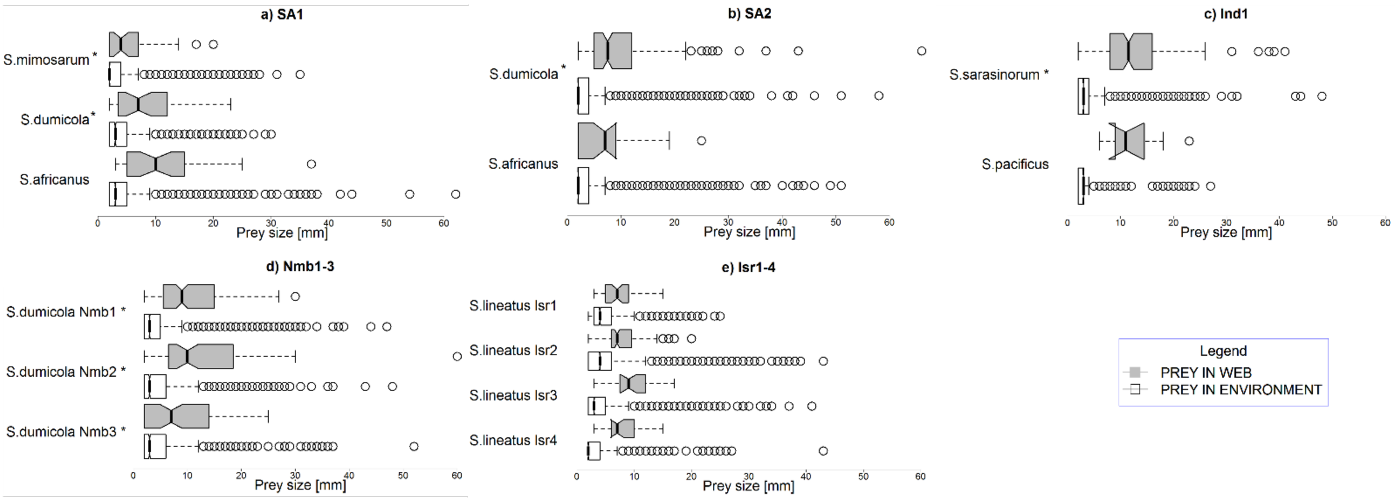

Figure S1. Boxplots of prey size captured by the webs (grey boxes) and in the adjacent traps (white boxes) at each of the studied sites. Co-occuring species are plotted in first row as follows: (a) solitary (*S. africanus*) and social (*S. mimosarum* and *S. dumicola*) in SA1; b) solitary (*S. africanus*) and social (*S. dumicola*) in SA2; c) solitary (*S. pacificus*) and social (*S. sarasinorum*) in Ind1. Single species are plotted in the second row: d) social *S. dumicola* at Nmb1-3, and e) solitary *S. lineatus* at Isr1-4. When comparing the prey caught in the webs of co-occuring species, the only statistically significant difference was detected for site SA1 (solitary *S. africanus* captured larger prey: mixed model sociality effect, ANOVA $F_{1,13}$= 24.85, p<0.001). Abbreviations for site names in the panel titles refer to sites in South Africa (SA1 and SA2), Namibia (Nmb), India (Ind1) and sites 1-4 in Israel (Isr). Each boxplot shows the extremes, the inter-quartile range, and the median. Social species are marked with *.

Supplementary material_ ***Cooperative foraging expands dietary niche but does not offset intra-group competition for resources in social spiders.*** Authors: Marija Majer, Christina Holm, Yael Lubin & Trine Bilde

Table S1. Results of ANOVA analyses of prey size as response variable. Size in traps and webs recorded in each location was analysed (glmm with negative binomial family and log link, trap and trap type were set as random effects). P-values are given in brackets if the statistic was significant.

**Species**	**Site**	**Method (web/trap)**	**Prey order**	**Collection date**	**I (Collection date*Prey.order)**	**I (Method*Prey.order)**
S. africanus	SA1	$F_{1,8}$=15.57 (0.004)	$F_{6,2359}$= 138.99 (0.001)	$F_{1,2359}$= 5.02 (0.03)	$F_{6,2359}$= 7.69 (0.001)	n.s.
	SA2	$F_{1,9}$=11.82 (0.009)	$F_{5,4576}$=497.22 (0.0001)	$F_{1,4576}$=10.16 (0.001)	$F_{5,4576}$=5.37 (0.0001)	$F_{5,4576}$=6.17 (0.0001)
S. lineatus	Isr1	$F_{1,28}$=36.70 (< .0001)	$F_{6,1682}$=36.42 (< .0001)	$F_{1,1682}$= 8.12 (0.004)	n.s.	n.s.
	Isr2	$F_{1,51}$=36.58 (< .0001)	$F_{4,4667}$=458.94 (< .0001)	$F_{1,4667}$=4.70 (0.03)	$F_{4, 4667}$=4.08 (0.003)	$F_{4, 4667}$=14.94 (<.0001)
	Isr3	$F_{1,14}$=75.88 (<.0001)	$F_{5,6128}$=268.35 (<.0001)	$F_{1, 6128}$=7.14 (0.008)	n.s.	n.s.
	Isr4	$F_{1,18}$=57.17 (< .0001)	$F_{4,1616}$=115.86 (< .0001)	$F_{1,1616}$=4.71 (0.03)	n.s.	n.s.
S. pacificus	Ind1	$F_{1,2}$=33.80 (0.03)	$F_{3,288}$= 126.69 (0.0001)	$F_{1,288}$=5.42 (0.02)	$F_{3,288}$= 8.96 (0.0001)	n.s.
S. dumicola*	SA1	n.s	$F_{6,1672}$=85.04 (<.0001)	n.s	$F_{6,1672}$=5.15 (0.0001)	n.s
	SA2	$F_{1,15}$=24.22 (0.002)	$F_{9,6711}$=194.07 (0.0001)	n.s	$F_{9,6711}$=2.94 (0.002)	$F_{9,6711}$=7.17 (0.0001)
S. dumicola*	Nmb1	$F_{1,17}$=70.23 (0.0001)	$F_{6,2732}$=306.57 (0.0001)	n.s	$F_{6,2732}$=4.58 (0.0001)	$F_{6,273}$2=10.45 (0.0001)
	Nmb2	$F_{1,17}$=68.96 (0.0001)	$F_{7,2285}$=117.88 (0.0001)	n.s	$F_{7,2285}$=3.96 (0.0003)	$F_{7,2285}$=11.88 (0.0001)
	Nmb3	$F_{1,13}$=9.21 (0.01)	$F_{7,2379}$=72.36 (0.0001)	$F_{1,2379}$=25.90 (0.0001)	$F_{7,2379}$=2.33 (0.02)	$F_{7,2379}$9=3.76 (0.001)
S. mimosarum*	SA1	n.s	$F_{5,3435}$= 177.31 (0.001)	$F_{1,3435}$= 18.81 (0.001)	$F_{5,3435}$= 6.19 (0.001)	n.s.
S. sarasinorum*	Ind1	$F_{1,39}$=200.07 (0.0001)	$F_{9,2315}$= 159.83 (0.0001)	n.s.	$F_{9,2315}$= 3.50 (0.0003)	$F_{9,2315}$= 4.30 (0.0001)

Supplementary material_ ***Cooperative foraging expands dietary niche but does not offset intra-group competition for resources in social spiders***. Authors: Marija Majer, Christina Holm, Yael Lubin & Trine Bilde

Table S2. Results of statistical comparisons of frequencies of different prey orders caught in webs vs. traps at each location. Presented here are Chi-square statistics and Spearman's rho correlation of standardized residuals of the frequencies of orders of prey caught in webs (Spearman's rho = *(observed count-expected count)/square root (expected count)*) to frequencies of the same prey orders caught in the traps (p-values are given in brackets, NA if sample size was too small). Degrees of freedom (df) refer to the number of different prey orders for which $\chi2$ was computed. The number differs between sites and species, since some prey types were caught only in traps and not in the webs, and vice versa. * = Social species.

**Species**	**Site**	**Prey number**		**Chi-square (p-value)**	**Spearman's rho (p-value)**
		WEB	TRAP	WEB *vs.* TRAP	
S. africanus	SA1	37	2830	$\chi2$= 62.15, df=8 (**<.0001**)	-0.5 (n.s.)
	SA2	26	5961	$\chi2$ = 58.77, df=8 (**<.0001**)	-0.33 (n.s.)
S. lineatus	Isr1	45	1728	$\chi2$= 20.24, df=7 (**0.005**)	-0.24 (n.s.)
	Isr2	155	6053	$\chi2$ = 121.3, df=7 (**<.0001**)	-0.52 (n.s.)
	Isr3	27	6502	$\chi2$ = 57.22, df=8 (**<.0001**)	-0.49 (n.s.)
	Isr4	31	2552	$\chi2$ = 30.67, df=8 (**.0001**)	-0.11 (n.s.)
S. pacificus	Ind1	7	299	NA	NA
S. dumicola*	Nmb1	188	3249	$\chi2$ = 222.96, df=8 (<**.0001**)	-0.15 (n.s.)
	Nmb2	95	2689	$\chi2$= 242.72, df=8 (<**.0001**)	-0.37 (n.s.)
	Nmb3	82	2802	$\chi2$ = 55.40, df=8 (<**.0001**)	-0.30 (n.s.)
S. dumicola*	SA1	35	1815	$\chi2$= 50.94, df=7 (<**.0001**)	-0.64 (n.s.)
	SA2	160	8166	$\chi2$= 143.27, df=8 (<**.0001**)	-0.67 (n.s.)

S. mimosarum*	SA1	38	3840	$\chi 2 = 35.07$, df=8 (<**.0001)**	-0.15 (n.s.)
S. sarasinorum*	Ind1	166	2323	$\chi 2 = 528.79$, df=8 (<**.0001**)	-0.63 (n.s.)

Table S3. Results of similarity analyses of abundance data on prey types caught by webs, sticky and window traps. Permutation manovas on distance matrices (computed on Chao and Morisita similarity indices) were used to assess the difference in abundances of different insect orders in relation to location, nest/trap height and species. R^2 measures apply to Chao and Morisita-Horn indices, respectively.

**Method**	**Effect**	**Pseudo F (p-value)**		**R^2 (Chao, Morisita-Horn)**
		Chao	*Morisita-Horn*	
**Web**	Species	$F_{4,145}$=5.21(0.001)	$F_{4,145}$=3.87(0.001)	0.12, 0.09
	Location	*n.s.*	*n.s.*	0.05, 0.05
	Nest height	$F_{1,145}$=2.22(0.07)	$F_{1,145}$=2.54(0.07)	0.01, 0.01
**Sticky traps**	Species	$F_{4,86}$=65.9(0.001)	$F_{4,86}$=57.2(0.001)	0.74, 0.57
	Location	*n.s.*	$F_{7,86}$=11.98(0.001)	0.02, 0.21
	Trap height	*n.s.*	*n.s.*	0, 0
**Window traps**	Species	$F_{4,86}$=31.54(0.001)	$F_{4,86}$=31.45(0.001)	0.59, 0.52
	Location	*n.s.*	$F_{7,86}$=4.04(0.001)	0, 0.12
	Trap height	*n.s.*	*n.s.*	0.01, 0

Supplementary material_ ***Cooperative foraging expands dietary niche but does not offset intra-group competition for resources in social spiders***. Authors: Marija Majer, Christina Holm, Yael Lubin & Trine Bilde

SUPPLEMENTARY ANALYSES: B. REGRESSIONS OF GROUP SIZE ON NEST VOLUME

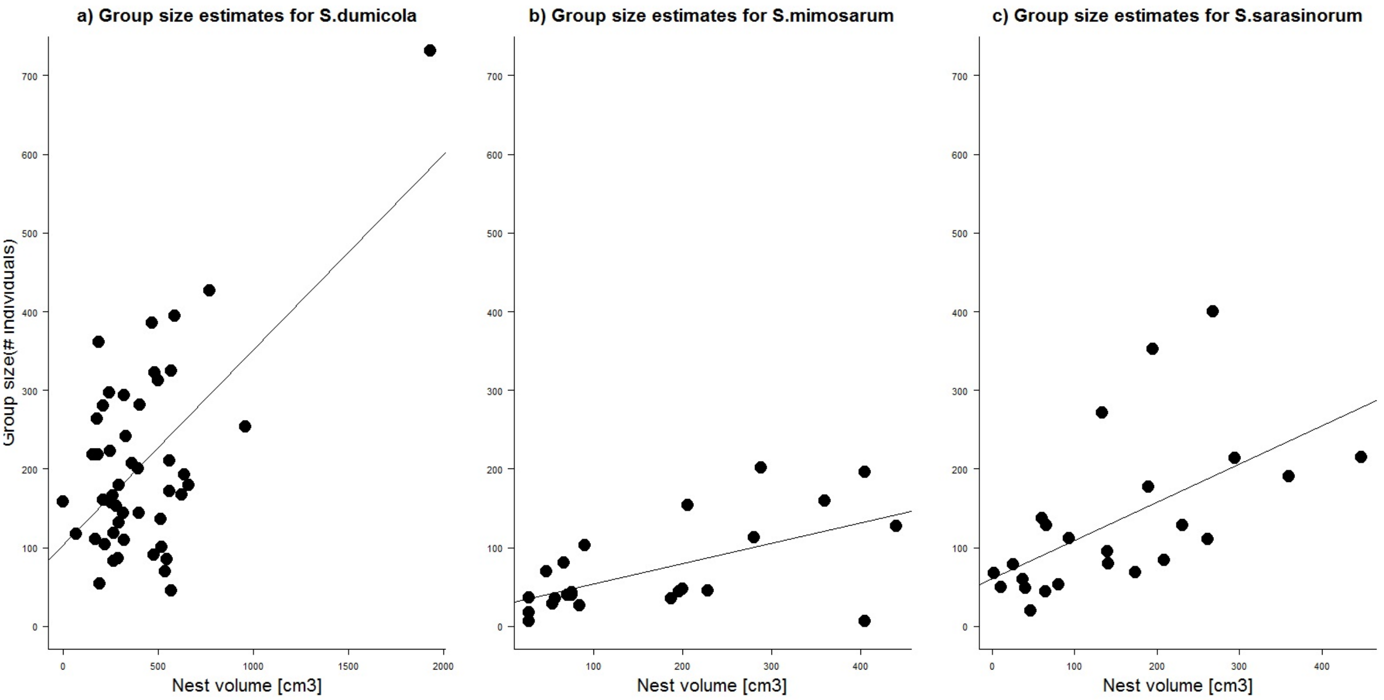

Figure S2. Raw data on nest volumes and group sizes for colonies of three social *Stegodyphus* species: *S. dumicola* (a); *S. mimosarum* (b) and *S. sarasinorum* (c). Fitted lines show linear regression fits for group size (y) as a function of nest volume (x). These regressions were used to derive the group sizes from measured nest volumes of colonies used in the studies presented in the main file. Derived regressions are y = 0.2475x + 103.76, R^2 = 0.35 for *S. dumicola*; y=0.1291x + 28.356, R^2 = 0.35 for *S. mimosarum;* y = 0.4068x + 47.671, R^2 = 0.69 for *S. sarasinorum*.

References

Anderson, M.J. A new method for non-parametric multivariate analysis of variance. *Austral ecology*, **26**, 32–46 (2001).

Chao, A., Chazdon L., R., Colwell K., R. & Shen, T.-J. *A new statistical approach for assessing similarity of*

species composition with incidence and abundance data (2005).

Wolda, H. Similarity indices, sample size and diversity. *Oecologia*, **50**, 296–302 (1981).
